# Monte Carlo approach to risks assessment of heavy metals at automobile spare part and recycling market in Ilorin, Nigeria

**DOI:** 10.1038/s41598-020-79141-0

**Published:** 2020-12-16

**Authors:** Muyiwa Michael Orosun, Abayomi Daniel Adewuyi, Naheem Banji Salawu, Matthew Omoniyi Isinkaye, Olugbenga Rapheal Orosun, Adetola Sunday Oniku

**Affiliations:** 1 Department of Physics, University of Ilorin, Ilorin, Nigeria; 2 Department of Geophysics, Federal University Oye-Ekiti, Ekiti, Nigeria; 3 Department of Physics, Ekiti State University, Ado-Ekiti, Nigeria; 4 Department of Electrical/Electronic Engineering, Bayero University, Kano, Nigeria; 5 Department of Physics, Modibbo Adama University of Technology, Yola, Nigeria

**Keywords:** Natural hazards, Environmental chemistry, Environmental impact

## Abstract

This study evaluates the sources and health risks associated with heavy metals in Ipata spare part market in Ilorin, Nigeria. Soil and water samples were collected within and outside the market for heavy metal (As, Pb, Mg, Mn, Cd, Cr, Cu, Zn, Fe and Ag) analysis using Atomic Absorption Spectrometry. The results indicate that all the heavy metals analyzed show higher concentration within the recycling market than the control location. The concentration of heavy metals at the market decreases with an increasing depth between 0 and 50 cm and appears to be stable below 50 cm of the soil depth. All the Hazard Indices (HI) estimated for the soil samples are less than one (< 1) which is the standard set by USEPA, whereas, the Hazard Index (HI) for the water samples within the station is greater than 1. The Incremental Lifetime Cancer Risk (ILCR) for soil samples ranged from level I to level V, while that of water samples ranged from level VI to Level VII based on Delphii method of classification. This shows that the main lifetime cancer risk occurs through the water exposure pathway. Similarly, according to the mean, P5% and P95% cumulative probability using the Monte Carlo simulation, the ILCR is above the acceptable range of 1.00E−6 and 1.00E−4. All the pollution indices reveal that the significant pollution at the park is more of anthropogenic than pedogenic and lithogenic. Therefore, the market is contributing immensely to environmental pollution which may lead to unforeseen danger to human health.

## Introduction

Africa is the den of automobile wastes. Fairly used and sometimes outdated cars from other parts of the world are exported to Nigeria and other African countries. Most of the vehicles imported into Nigeria for instance, are in bad shape and not road worthy^1,2^. Up to the time of writing this paper, statutory laws necessary to regulate the quality or quantity of vehicles to be imported are not effectively enforced because most of the vehicles are smuggled into the country via the porous border. Additionally, lack of technology and functional system to manage the resulting automobile wastes will continue to cause enormous volumes of these wastes to be pilling up at automobile stations in worrying rate.

Recent investigations revealed that particles emitted at automobile spare part and recycling parks are accompanied with growing metal concentrations (principally Zn, Pb, Cd and Cu on the account of decommissioning and abandonment, dismantling, welding, paints, grease, fuel additives, tires and brake dust, poor disposal and rusting of spare parts), causing serious soil pollution. These anthropogenic activities enhance the levels of heavy metals (HMs) in the environment which may pose a potential human health hazard^3,4^.

Heavy metals (HMs) also referred to as potential toxic elements (PTE), are nuclides whose specific gravity or density is at minimum five times that of water. These elements have their relative atomic mass ranging between 63.546 and 200.590 u (atomic mass unit) and are detrimental to human health once they exceed normal concentration in the human body. This classification may include elements of groups III to V of the periodic table, actinides and lanthanides, transition metals and some metalloids^5^. Some examples are Hg, Zn, Mn, Mg, As, Pb, Cr, Cd, Ni, Co, Cu, Bi, Fe, etc. HMs are natural constituents of the Earth and this has led to human exposure in one way or the other throughout the whole history of mankind^6,7^.

These elements when introduced into the environment by activities like the one taking place at Ipata Oloje automobile spare part and recycling market, usually find their way into human bodies to an extent via ingestion of soil (dust) particle, through food chain, drinking of contaminated water and dermal contact^8–10^. Some of these HMs are vital to human life as sources of minerals and vitamins, and/or play irreplaceable physiological roles in the human body, but become toxic at higher concentration and can therefore result to poisoning at relatively high concentrations^6^. This poisoning may result from inhaling air of high concentration near emission sources, drinking-water contamination, or ingestion via food chain. The terrible thing about these heavy metals in the human body is that they tend to bio-accumulate. If these HMs accrue in the tissues faster than the body’s detoxification rate, there would be a gradual build-up of these toxins^11^. Bioaccumulation is the gradual build-up of chemicals in living organism with respect to the chemical’s concentration in the environment over time. Any time compounds are ingested, stockpiled quicker than they are used up or excreted, these compounds end up being accumulated in the body^12,13^.

The presence of these HMs in the human body can result to severe health effects with different symptoms, depending on the type and concentration of the metal ingested^14^. Toxicity of HMs is formed by the creation of complexes whenever they interact with proteins, in which amine (–NH_2_), the carboxylic acid (–COOH), and the thiol (–SH) groups are majorly involved. High concentration of heavy metal in the body also affects protein structure, which is connected to the catalytic properties of enzymes. Most important enzymes are made dormant whenever they bind to this group of metals. The altered biological molecules stop functioning properly and may result to the death of the cells. This type of toxin is as well responsible for the creation of radicals which are hazardous chemicals that cause oxidation of biological molecules^15^.

Sufficient protection and restoration of our environments contaminated by HMs require characterization of their sources and nature, and providing remediation. Existing legislations in respect of public health and environmental protection worldwide are based on researches that characterize biochemical properties of environmental phenomena, particularly the ones that exist in our waterways and food chain^16,17^. Even though soil characterization would offer insight into bioavailability and speciation of HMs, effort at remediation of soils contaminated by HMs would require knowledge of the source of contamination, chemistry, and assessment of the associated health risks of the HMs (Carcinogenic and Non-carcinogenic risks). More so, risk assessment has been proven to be an effective scientific contrivance which enables decision makers and law enforcers to manage sites so contaminated in a cost-effective modus while conserving the ecosystem and public health^18,19^.

## Materials and methods

### Location and geology of the study area

**Figure 1:**
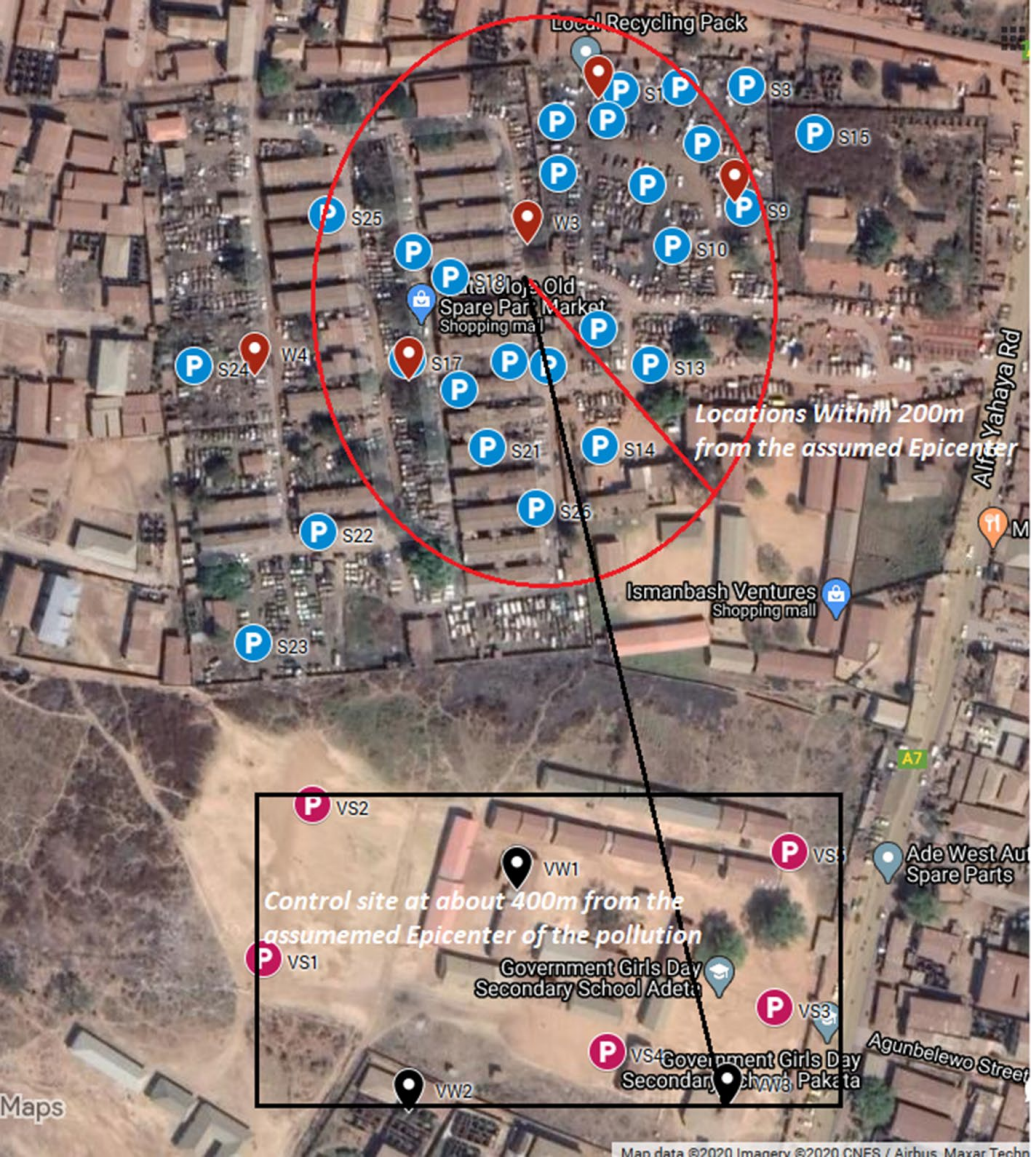
Satellite map of the study area showing sample locations (Available at: https://www.google.com/maps/d/u/0/edit?mid=1Umhqso1frIWVxV5aIrvMLoJXbHDGf4AO).

The study area is Ipata Oloje automobile spare part and recycling market, Ilorin, Nigeria (Fig. 1). It is located between Longitudes 4° 25′ E and 4° 65′ E and latitudes 8° 20′ N and 8° 50′ N, having a geographic region of about 100 km^2^. According to National Population Commission of Nigeria, the population of the study area is 205,567 as at 2006 Census and projected to be 365,221 in 2016. Area: 105 km^2^—Density: 4695/km^2^ at + 3.05%/year increment. 43.5% of these populations are children between 0 and 14 years, 53.3% are between 15 and 64 years and 3.2% are older people above 64 years^20^. On the geology of the study area, Ilorin consists of Pre-Cambrian basement complex with elevation that ranges between 273 and 333 m in the West harboring an isolated hill called Sobi hills which is about 394 m above sea level, and ranges between 200 and 364 m in the East^4,21,22^. Some part of Ilorin town is reportedly underlain by sedimentary rock, which consists of laterites and alluvial deposits^23^. There is a large number of ferruginous groups of soils majorly because of the different species of basement complex rocks. Thus, the soil in Ilorin is mainly the ferrallitic type, having a deep red colouration with great clay content. The soils originate from the metamorphic and igneous rocks (basement complex rocks) which is nearly 95%. The quartzite augitegnesiss, banded gnesiss, biotite gnesiss, and granitic gnesiss are the major constituents of the metamorphic rocks^21–26^.

#### Description of the automobile spare part and recycling market

The Ipata Oloje automobile spare part and recycling market is characterized by several activities like decommissioning and abandonment, dismantling, welding, paintings, poor disposal and rusting of spare parts. Most of the badly shaped cars imported into the country are dismantled into parts for sale. This includes accidental vehicles whose engines and other parts are still useful. While the spare part and recycling market is famous for dismantling and selling of spare parts, repairs (general servicing, engine repairs, panel beatings and welding, painting, greasing, fuel additives, tires and brake fixing etc.) of fairly damaged vehicles is one of the dominant activities taking place in this market. The most worrying sight is how the inhabitants coexist within the spare part and recycling market. You cannot separate residential buildings from the market as the activities of the market has spread over the places. This spread makes this spare part and recycling market by far the biggest in North-central Nigeria. From Fig. 1, inhabitants living within 200 m from the assumed epicenter of the pollution source are believed to be the most vulnerable. As such, samples of soil were randomly collected and water samples were also collected from already constructed wells that were accessible. Areas around Government Girls Day Secondary Schools about 400 m away were considered unpolluted and therefore used as the control samples because it is far from the automobile spare part and recycling market and free from pollution but share same local geology with the market.

#### Classification of the automobiles

On the basis of load or capacity, the automobile spare part and recycling park consists of mainly light transport vehicle (LTV) or light motor vehicle (LMV) which carries light stuffs and is smaller in size (examples includes passengers cars like sedans, saloons, min-vans, vans, sport utility vehicles etc.). On the basis of wheels and fuel type used, these automobiles are four wheels vehicles and mostly the ones that use petrol (i.e. petrol vehicles not diesel vehicles, steam vehicles or electric vehicles).

### Sample collection and preparation

#### Collection and preparation of soil samples

Twenty-six (26) samples of the top soil were taken randomly within the automobile spare part and recycling park. Five (5) samples of the top-soil were taken randomly outside the study area (i.e. locations around the Government Girls Day Secondary Schools Adeta, about 400 m away site) considered as unpolluted and therefore used as the control samples. Six (6) samples of soil were also collected at every 10 cm of the soil depth to 100 cm (vertical distance) within the automobile spare part and recycling park. The soil samples were obtained in suitable test containers (polyethylene plastics) of about 10 cm^3^ each using a soil auger during the dry season between December 2018 and March 2019. These samples were then taken to Chemistry Laboratory, University of Ilorin, where macroscopic traces of stones, plastic rubbers, glass, animal and plant matter and other large particles were removed to ensure that the materials to be analyzed are free from such impurities. The samples were then dried using air at room temperature in the Laboratory for 14 days to lessen the mass contribution of water and to inhibit any chemical reaction^27^. The samples were crushed with agate mortar, sieved through a 1 mm sieve mesh and then stored in fit labeled plastic containers for digestion. For the digestion of trace metals in soil samples, method of Aqua Regia was used. 1 g of the soil sample was weighed into a hygienic digestion flask and 3 ml of concentrated HNO_3_, 9 ml of concentrated HCl were added into the sample in the digestion flask^28^. The mixtures were heated until it stops giving off brown fumes (denoting the release of Nitrogenous compounds) which confirms completion of digestion. The samples were allowed to cool and a few drops of distil water were added and the mixture was filtered into 25 ml standard flask which was transferred into plastic reagent bottle for Atomic Absorption Spectrometry (AAS) which is for the quantitative determination of the concentrations of elements of interest in a given sample. The technique gives the concentrations of the heavy metals in the digested samples up to parts per million. The elemental analysis was done at ROTAS Soil-Lab Ibadan, Nigeria using Buck Scientific Atomic Absorption Spectrophotometer Model 210 VGP (Buck Scientific, E. Norwalk, CT, USA).

#### Collection and preparation of water samples

A total of 8 wells that are accessible were taken into consideration (5 wells within the study area and 3 control samples). 3 water samples each were obtained in a suitable rubber test container from each of the wells. The accessible wells are the ones used by the general public. These samples were labeled for easy identification. The water samples from the study area were collected in hygienic polyethylene bottles. The water samples were filtered through a 0.45 µm membrane filter immediately after collection. For the digestion, 100 ml of the water samples were measured into a clean dry digestion flask. 3 ml of concentrated HNO_3_ and 9 ml of concentrated HCl were added into the sample in the digestion flask^28,29^. The solutions were heated until all the brownish fumes (Nitrogenous Compound) were given off confirming that the digestion of the samples is complete. The samples were then allowed to cool at normal room temperature. A few drops of distilled water were then added and the mixtures were filtered into 25 ml standard flask which was later transferred into rubber reagent bottle (polyethylene plastics) for Atomic Absorption Spectrometry (AAS).

#### Atomic absorption spectrometry set up and analysis

Aqua-Regia method of digestion was employed to digest the samples for the elemental analysis using Atomic Absorption Spectrometry as stated earlier. The concentrations of the selected heavy metals was determined using Buck Scientific Atomic Absorption Spectrophotometer Model 210 VGP (Buck Scientific, E. Norwalk, CT, USA) at ROTAS Soil-Lab Ibadan, Nigeria. The machine parameters are given in Table 1. All reagents used were resolved into elements or their constituent parts. Working standards of lead, cadmium, chromium, arsenic, manganese, magnesium, copper, silver, zinc and iron were prepared by mixing concentrated stock solutions (Merck, Germany) of 1000 ppm with ultra-pure water (MilliQ, Millipore-USA)^4,30^. A calibration curve was plotted for each element employing the measured absorbance value for the blank and working standard solution so as to estimate the concentrations of heavy metals in the samples digested. Blank samples were used to cancel-out the background effects of the reagents and distilled water as well as calculate the detection limit of the analyzing instruments. The detection limits of instrument varied from 0.005 ppm (Cu, Ag, Mg and Zn) to 0.080 ppm (Pb). To ensure quality control, standard procedures were followed, the samples were handled carefully and all the vessels utilized (i.e. glass wares and digestion vessels) were washed thoroughly before use, rinsed and purified with de-ionized water. Precision and accuracy of the measuring procedures were made certain through the reagent blanks and duplicate samples preparation^31,32^.

**Table 1:** Background information of the buck scientific atomic absorption spectrophotometer.

Elements	Detection limits mg/L	Sensitivity check mg/L	Wavelength Nm	Linear range	Slit Nm
Fe	0.050	2.5	248.3	5.0	0.2
Cu	0.005	2.0	324.8	5.0	0.7
Pb	0.080	10.0	283.3	20	0.7
Mn	0.030	1.25	279.5	2.50	0.7
Mg	0.005	0.75	285.2	1.50	0.7
As	0.050	3.5	422.7	6.0	0.7
Zn	0.005	0.5	213.9	2.50	0.7
Cd	0.010	0.75	228.9	2.0	0.7
Cr	0.040	2.0	357.9	5.0	0.7
Ag	0.050	0.5	328.1	2.0	0.2

### Pollution evaluation

#### Modified enrichment factor (MEF)

The Enrichment factor presents a suitable measure of geochemical trend and enhancement. The enrichment of the HMs was quantified using the modified enrichment factor (MEF) given by Eq. (1)^33,34^:

1 $${\text{MEF}} = \frac{{\left( {\frac{{C_{i} }}{{C_{ref} }}} \right)_{Sample} }}{{\left( {\frac{{C_{i} }}{{C_{ref} }}} \right)_{Control} }}.$$

where C_i_ and C_ref_ are the concentration of the target (usually concentration of HMs in the polluted samples) and reference elements (mean concentration of HMs in the control samples) respectively^33^. T results were classified in accordance with^33^ where EF (< 2) values corresponds to minimal enrichment, values (2–5) = moderate enrichment, (5–20) = significant enrichment, (20–40) = very high enrichment and values (> 40) = extremely high enrichment.

#### Modified Pollution Index (MPI)

The modified pollution index (MPI) was quantified using Eq. (2). The MPI presents convenient and reliable method of assessing the degree or amount of contamination of a given sample of soil using the MEF values^33^.

2 $${\text{MPI }} = \sqrt {\frac{{\left( {MEF_{mean} } \right)^{2} + (MEF_{Max} )^{2} }}{2}}$$

The following terms were used for the MPI based on values: MPI < 1, unpolluted; 1 < MPI < 2, slightly polluted; 2 < MPI < 3, modately polluted; 3 < MPI < 5, significantly polluted; 5 < MPI < 10, severely polluted; and MPI > 10, extremely polluted.

#### Quantification of anthropogenic metal (AM)

Assume that the concentration of the HMs in the control samples is adopted to represent lithogenic metal content, the anthropogenic metal (AM) was estimated for each HM using Eq. (3) described by^34^.

3 $${\text{AM }} = \frac{{ C_{ sample} - C_{ Control } }}{{C_{Control} }} \times 100{\text{\% }}$$

where C_control_ = concentration of the HMs at the control site representing the lithogenic HMs content. And C_sample_ = the mean concentration of the HMs in the contaminated soil.

### Health risk assessment

Human health risk assessment is a procedure usually employed in estimating the related health effects that may possibly result from exposure to carcinogenic and non-carcinogenic chemicals. There are four main steps involved in the risk assessment process: hazard identification, assessment of exposure, toxicity/dose–response assessment, and then characterization of risk^35^. The aim of hazard identification is to basically examine pollutants that are present in a specified location, their concentrations, and spatial distribution. Assessment of exposure is mainly to evaluate the intensity, frequency, and length or period of human exposures to the contaminants (i.e. HMs). In this research, the assessment of exposure was done by calculating the average daily intake (ADI) of the HMs identified through ingestion, inhalation and dermal contact by the inhabitants. Dose–response assessment evaluates the toxicity due to exposure intensities of the HMs. A carcinogen potency factor known as cancer slope factor (SF), and a non-carcinogenic threshold called reference dose (R_f_D), are the two vital toxicity indices employed. Risk characterization helps predicts the probable cancerous and non-cancerous health risks the general populace in the study area are exposed to, by incorporating all the information collected to work out quantitative estimates of cancer risks and hazard indices.

The average daily intake (ADI) (mg/kg/day) for non-carcinogens through ingestion, inhalation and dermal contact exposure pathways were calculated using Eqs. (4)–(8) recommended by^35^.

For ingestion pathway,

4 $${\text{ADI}}_{{\text{ing-soil}}} = { }\frac{{Cs \times { }IngRs \times EF \times {\text{ ED}}}}{BW \times AT}$$

5 $${\text{ADI}}_{{\text{ing-water}}} { } = { }\frac{{Cw \times { }IngRw \times EF \times {\text{ ED}}}}{BW \times AT}$$

where ADI_ing-soil_ and ADI_ing-water_ are the average daily intake of heavy metals from soil and water ingestion (mg/kg-day) respectively, Cs and C*w* are the concentration of heavy metal in soil and water sample, BW is body weight of the exposed individual, ED is the lifetime exposure duration (year), IngRs and IngRw are the ingestion rate of soil and water particles (mg/day or L/day) respectively, EF is the exposure frequency (day/year), and AT is time period over which the dose is averaged (day).

For inhalation pathway,

6 $${\text{ADI}}_{{\text{inh - soil}}} = \frac{{Cs \times InhRs \times { }EF \times { }ED}}{{PEF \times BW \times { }AT}}$$

where PEF is the particle emission factor (m^3^/kg).

For Dermal pathway,

7 $${\text{ADI}}_{{{\text{derm}}}} = \frac{{C \times { }SA \times { }AF{ } \times { } ABS \times { }EF \times { }ED}}{{BW \times { }AT}}$$

8 $${\text{ADI}}_{{\text{derm - water}}} = \frac{{C \times { }SA \times { }KP \times { }AF{ } \times { } ABS \times ET \times { }EF \times { }ED}}{{BW \times { }AT}}$$

where SA is the exposed skin surface area (cm^2^), KP is the permeability constant of the skin, ABS is the skin absorption factor, ET is the exposure time.

#### The non-carcinogenic risk assessment

Target Hazard Quotient (HQ) which is the ratio of the protracted average daily intake (ADI) to the reference dose (RfD) of a particular heavy metal (HM)^35^, is used to estimate or assess the non-carcinogenic risk. The target hazard quotient (THQ) adopts a level of exposure called the reference dose (RfD), which is known as the daily absorption rate that is projected to have no significant risk of adverse health effects, over about 70-years lifetime. The formula is given by USEPA to be;

9 $${\text{THQ}} = \frac{ADI}{{RfD}}$$

where ADI is the average daily intake of a single toxic element and RfD is the chronic reference dose for the element (mg/kg-day)^13,35^. If the target hazard quotient is greater than 1, then there’s heavy likelihood of adverse health effect to the exposed population. However, if the target hazard quotient is less than 1 then there’s no likelihood of adverse health effects.

The hazard index (HI) is defined as the total sum of HQ obtained for different pathways as shown in Eq. (10). To evaluation the human health risks through more than a single heavy metal, the hazard index (HI) was established as^35^:

10 $${\text{HI}} = \sum HQ$$

#### The carcinogenic risk assessment

The carcinogenic risk assessment is used in the estimation and determination of the possibility of a population acquiring cancer of any kind after exposure to carcinogen. Incremental Lifetime Cancer Risk (ILCR**)** is estimated as the incremental probability of a person developing cancer over a period of time due to exposure to HMs^6,31^. The formula is given as;

11 $${\text{ILCR }} = {\text{ADI }} \times {\text{ SF}}$$

where ILCR is the probability of an individual exposed to carcinogenic HMs to develop cancer over a period of time. ADI (mg/kg/day) and SF (mg/kg/day) are the average daily intake and the carcinogenic slope factor respectively. For cancer risk, only the known human carcinogens (Pb, Cr, Cd, and As) were considered. Cancer risk greater than $1 \times 10^{ - 4}$ are considered high as they pose higher cancer risk while values below $1 \times 10^{ - 6}$ are considered not to pose any cancer risk to humans; the acceptable range is between $1 \times 10^{ - 4}$ and $1 \times 10^{ - 6}$. The risks values are categorised in to 7 levels based on the Delphii method according to^36,37^ and are given in Table 2.

**Table 2:** Levels and values of assessment standards^36,37^.

Risk levels	Range of risk value	Acceptability
Level I (extremely low risk)	< 10^–6^	Completely accept
Level II (low risk)	10^–6^, − 10^–5^	Not eager to care about the probable risk
Level III (low-medium risk)	10^–5^, − 5 × 10^–5^	Not to be mindful about the risk
Level IV (medium risk)	5 × 10^–5^, − 10^–4^	Worry about the probable risk
Level V (medium–high risk)	10^–4^, − 5 × 10^–4^	Care about the risk and willing to invest
Level VI (high risk)	5 × 10^–4^, − 10^–3^	Pay attention and take action to solve it
Level VII (extremely high risk)	> 10^–3^	Must solve it

#### Monte Carlo simulation (MCS) using ORACLE crystal ball

The weight of a person (body weight), ingestion rate of the substance per day (ingested by an individual), concentration of the pollutant in the samples at the automobile spare part and recycling park and the carcinogenic slope factor of the pollutant, are all sources of uncertainty, which makes the evaluation of risk assessment a bit complicated. While overestimation of the health risk can cause waste of resources on needless remediation exercise, underestimation of the health risk can cause severe health consequence to the people living around the automobile spare part and recycling park. Evaluation of the mean and/or peak risk values using the health risk assessment model, either overestimates or underestimates the real risk^39^. The concern in estimating the risk assessments without simulation is that it is impossible to determine the probability (either above or below the 95th percentile) that a population will be at risk. Consequently, probabilistic approach using Monte Carlo simulation (MCS) has been appropriately employed in this research to assess more realistic risk related to chemical pollutants.

The Monte Carlo simulation has the advantage of minimizing uncertainty. In this method, arbitrary values are continually picked from the probability distribution of numerous values inputted to find the probability distribution of risk^39–41^. Rather than utilizing one-point value, in the MCS, several values are utilized to repeatedly calculate and lastly obtain the results with different assurance levels ranging from 1 to 99%. As stated earlier, many authors have employed this probabilistic approaches to inspect the probable harmful risks of chemicals in food, water and other environmental parameters. The software used in this work to perform the MCS is Oracle Crystal Ball software version 11.1.2.4.850.

## Results and discussions

The results of the heavy metal analysis carried out on the soil and water samples collected from within and outside the study area are given in Tables 3, 4, 5 and 6. The results are presented alongside the current drinking water quality guidelines for the selected heavy metals published by several organizations, committees or agencies throughout the world.

**Table 3:** Concentration of HMs in the selected top-soil within the study area in ppm.

S/N	Mg	M n	Ag	Zn	Cd	Pb	Cu	Fe	As	Cr
Min	42	58	2	105	8.2	25	18	340	35	90
Max	64	72	5	210	12	37	28	410	50	122
Median	54	68	3.5	162.5	10.2	28.8	23	380	42.5	108
Mean	53.7	66.7	3.5	154.7	10.05	28.79	22.6	378	42.48	108
SD	8.97	5.1	1.6	35.0	1.17	3.05	3.29	22.28	3.84	8.30
CV	16.78	7.57	45.14	23.24	10.90	10.51	14.75	6.04	9.13	7.83
WA	–	488.00	–	70.00	0.41	27.00	38.90	–	6.83	59.50
MAC	–	–	–	100–300	1–5	20–300	60–150	–	15–20	50–200
TAV	–	–	–	200–1500	2–20	50–300	60–500	–	10–65	50–450

*WA* world/global average for background contents, *MAC* maximum allowable concentration in soil, *TAV* trigger action value^4,43–45^.

**Table 4:** Concentration of HMs in the selected top-soil of the control site in ppm.

S/N	Mg	Mn	Ag	Zn	Cd	Pb	Cu	Fe	As	Cr
Min	4.06	3.4	ND	7.4	3.5	1.88	1.08	21.20	3.00	5.00
Max	4.82	4.0	2	8.8	4.2	1.98	1.24	24.20	3.50	6.00
Mean	4.30	3.6	0.8	8.1	3.8	1.94	1.14	22.46	3.34	5.80
SD	2.76	0.23	1.0	0.45	028	0.37	0.65	9.83	2.05	3.48
WA	–	488.0	–	70.00	0.41	27.00	38.90	–	6.83	59.50
MAC	–	–	–	100–300	1–5	20–300	60–150	–	15–20	50–200
TAV	–	–	–	200–1500	2–20	50–300	60–500	–	10–65	50–450

*ND* not detectable, *WA* world/global average for background contents, *MAC* maximum allowable concentration in soil, *TAV* trigger action value^4,43–45^.

**Table 5:** Mean concentration of HMs with depth within the station in ppm.

Depth (cm)	Mg	Mn	Ag	Fe	Zn	Cd	As	Cr	Pb
0.00	64.0	85.0	5.0	457.5	210.0	11.5	55.0	110.0	28.0
10.00	64.0	70.0	5.0	425.0	186.0	10.6	52.0	104.0	26.5
20.00	64.0	75.0	5.0	320.0	165.0	9.5	42.0	75.0	27.0
30.00	60.5	70.0	5.0	245.0	105.0	6.5	30.0	62.0	24.0
40.00	54.0	80.0	5.0	355.0	65.0	6.0	28.0	52.0	13.0
50.00	53.5	85.0	5.0	360.0	30.0	5.0	25.0	23.5	11.5
60.00	53.3	80.0	5.0	355.0	30.0	5.0	22.0	25.0	11.0
70.00	51.5	75.0	5.0	355.0	32.0	5.5	20.0	22.0	10.0
80.00	51.5	70.0	5.0	350.0	32.0	5.0	22.0	23.0	8.5
90.00	52.0	70.0	5.0	340.0	30.0	4.0	22.0	25.0	8.0
100.00	51.5	70.0	5.0	345.0	32.0	4.0	22.0	23.0	8.5
Min	51.5	70.0	5.0	345.0	30.0	4.0	22.0	22.0	8.0
Max	64.0	85	5.0	457.5	210.0	11.5	55.0	110.0	28.0
Overall mean	56.35	75.45	5.0	355.23	83.36	6.6	30.91	49.5	16
SD	5.53	6.11	0.0	53.95	71.06	2.67	12.76	33.88	8.40

**Table 6:** Mean concentration of HMs in well-water samples obtained within the automobile spare part and recycling park in ppm.

Well-water	Mg	Mn	Fe	Zn	Ag	Cd	As	Cr	Pb	Cu
Mean W1	1.35	0.06	1.30	2.50	ND	0.25	1.50	1.40	2.00	1.10
Mean W2	0.71	0.07	2.50	1.57	ND	0.18	2.22	2.70	1.20	2.18
Mean W3	0.76	0.06	1.52	1.50	ND	0.25	2.20	1.90	1.80	1.12
Mean W4	1.11	0.08	1.60	3.00	ND	0.20	0.58	2.50	1.60	1.16
Mean W5	1.50	0.08	1.85	2.20	ND	0.22	1.80	1.75	1.75	1.32
Min	0.71	0.06	1.30	1.50	ND	0.18	0.58	1.40	1.20	1.10
Max	1.50	0.08	2.50	3.00	ND	0.25	2.22	2.70	2.00	2.18
Overall mean	1.09	0.07	1.75	2.15	ND	0.22	1.66	2.05	1.67	1.38
SD	0.35	0.01	0.46	0.63	ND	0.03	0.67	0.54	0.30	0.46
WHO	–	0.10	0.30	3.00	–	0.03	0.01	0.05	0.01	2.00
USEPA	–	0.05	0.30	5.00	–	0.05	0.01	0.10	0.015	1.30
ECE	–	0.05	0.20	–	–	0.05	0.01	0.05	0.01	2.00

Detection Limit for Ag = 0.050 ppm.

*ND/NA* not detectable/available, *WHO* World Health Organization^46^, *USEPA* United Stated Environmental Protection Agency^47^, *ECE* European Commission Environment^48^.

### Concentration of heavy metals in the selected top soil within and outside the study area

**Figure 2:**
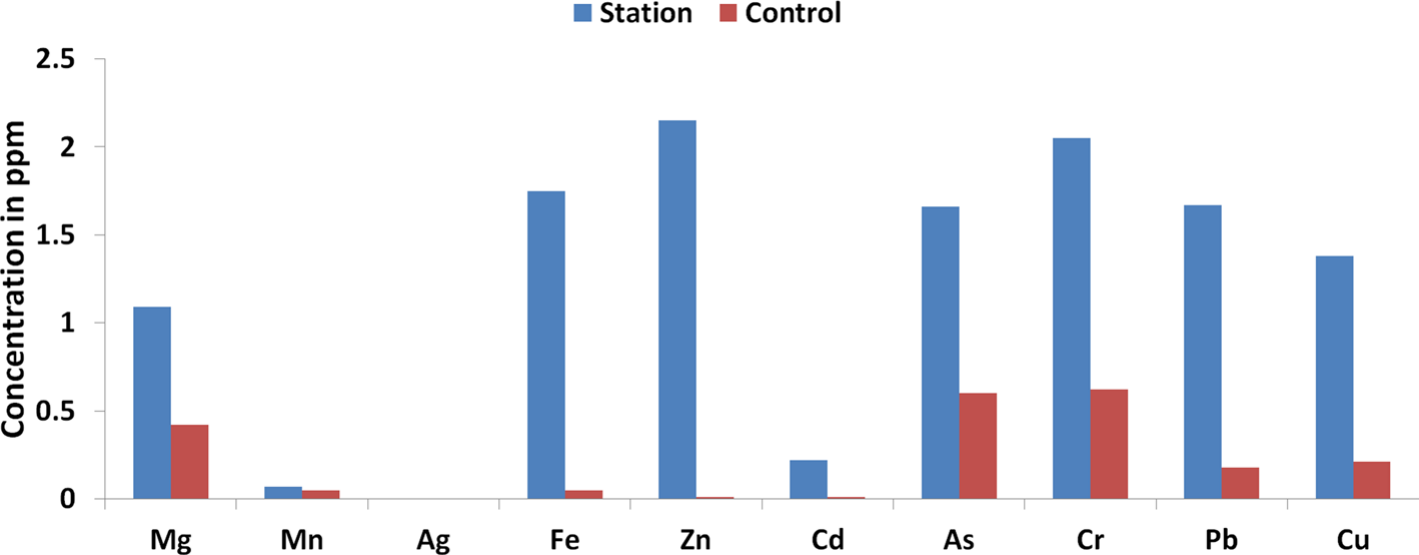
Graph of the mean concentration of heavy metals in the top soil within the automobile station and control site.

Table 3 presents the results of the geochemical analyses of heavy metals concentrations for the topsoil samples randomly obtained within the study area. Fe, Zn and Cr had higher concentration compared to the remaining elements (see Fig. 2). Despite the elevated levels of the concentration of these heavy metals, their mean values still fall below the Maximum Allowable Concentration (MAC) and Trigger Action Value (TAV) in soil, except Cadmium (Cd) whose reported mean value exceeds the MAC and TAV (see Table 3). The average value of the HMs in the selected top-soil is in the order: Fe > Zn > Cr > Mn > Mg > As > Pb > Cu > Cd > Ag. The relatively high concentration of Fe, Zn, and Cr was believed to be due to the gradual accumulation over time from various anthropogenic pollution sources related to the activities at the automobile spare part and recycling park (i.e. welding, discharges and dusts, poor disposal of automobile parts, painting, etc.). Higher values of Fe and Mn in the study area seem to have been mainly influenced by metallurgical sources, such as iron, steel and poor disposal of automobile spare parts. The observed enhanced values of Zn and Cu could also be associated with the activities at the automobile spare part and recycling park, because it may possibly result from deterioration of vehicular parts. Moreover, Zn is frequently used in the tyre production and Cu is a common element in vehicle thrust bearing, brake lining and other parts of the automobile engine^4,16,27^. Zinc compounds are effusively employed as anti-oxidants, as well as agents for improving dispersant for automobile oils^42^.

Geochemical analysis was also done for the top-soil samples randomly collected outside the study area (control site) and the results were presented in Table 4. It follows that the mean concentration of the selected heavy metals are lower than their respective concentrations at the study area.

From Table 5, the concentration of HMs in the automobile spare part and recycling park varies (decreases) with an increase in depth between 0 and 50 cm and appears to be stable below 50 cm of the soil depth at the park. Thus, it can be concluded that HMs at the study site is majorly due to the anthropogenic activities at the station (i.e. vehicular discharges and dusts, painting, wielding, poor disposal of spare parts etc.). This agrees with our earlier suggestion that the higher concentration of the heavy metals at the park is of anthropogenic rather than pedogenic and lithogenic sources^4,27^.

### Concentration of heavy metals in the water samples within the automobile spare part and recycling park and the control site

Because of the noticeable presence of contamination (pollution) from the analysis of the soil samples, the need to investigate the water samples within and outside the automobile spare part and recycling park is therefore very essential. The results of the heavy metal analysis for the well-water samples collected at the park as well as outside the park given in Tables 6 and 7 reveal higher mean concentrations of these pollutants at the automobile spare part and recycling park. This could readily be attributed to the anthropogenic inputs from the automobile spare part and recycling park. In comparison with the existing drinking water quality guidelines for the designated HMs, issued by renown agencies around the world given in Tables 6 and 7, it is evident that within the studied area, overall mean values of Magnesium (Mg), Manganese (Mn), Silver (Ag), Zinc (Zn) and Copper (Cu) are relatively lower than their respective maximum permissible concentration in drinking water. But Lead (Pb), Cadmium (Cd), Iron (Fe), Arsenic (As) and Chromium (Cr) clearly have mean concentrations at the automobile spare part and recycling park higher than the maximum permissible concentration in drinking water.

**Table 7:** Mean concentration of HMs in well-water samples collected from the control site in ppm.

Well-water	Mg	Mn	Ag	Fe	Zn	Cd	As	Cr	Pb	Cu
Mean V1	0.53	0.06	ND	0.03	0.01	0.01	0.80	0.80	0.25	0.20
Mean V2	0.31	0.02	ND	0.07	0.01	0.01	0.50	0.20	0.12	0.21
Mean V3	0.42	0.06	ND	0.05	0.01	0.01	0.50	0.85	0.16	0.21
Min	0.31	0.02	ND	0.03	0.01	0.01	0.50	0.20	0.12	0.20
Max	0.53	0.06	ND	0.07	0.01	0.01	0.80	0.85	0.25	0.21
Overall mean	0.42	0.05	0.00	0.05	0.01	0.01	0.60	0.62	0.18	0.21
SD	0.11	0.02	0.00	0.02	0.00	0.00	0.17	0.36	0.07	0.01
WHO	–	0.10	–	0.30	3.00	0.03	0.01	0.05	0.01	2.00
USEPA	–	0.05	–	0.30	5.00	0.05	0.01	0.10	0.015	1.30
ECE	–	0.05	–	0.20	-	0.05	0.01	0.05	0.01	2.00

Detection Limit for Ag = 0.050 ppm.

*ND/NA* not detectable/available, *WHO* World Health Organization^46^, *USEPA* United Stated Environmental Protection Agency^47^, *ECE* European Commission Environment^48^.

**Figure 3:**
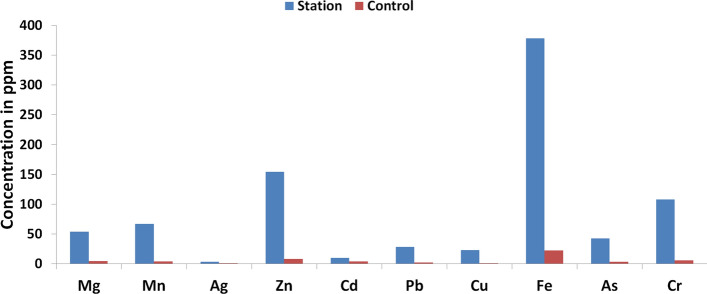
Graph of the concentration of Heavy Metals in the water samples within Automobile automobile spare part and recycling park and control site.

Although the overall mean values of the selected elements outside the automobile spare part and recycling park are less than the values recorded at the automobile spare part and recycling park (see Fig. 3), the mean values of Cr, Pb, and As are still above their respective recommended limits for consumption. This higher mean concentration in the water samples agrees with results of the soil analysis i.e. a common anthropogenic source relating to activities at the automobile spare part and recycling park (e.g. welding, painting, vehicular discharges and dusts, poor disposal of spare part, etc.).

### Principal component analysis (PCA) of heavy metals in soil samples

Principal Component Analysis (PCA) subject to Varimax rotation with Kaiser Normalization was carried out in order to ascertain the variations among the various heavy metals in the soil samples collected from the Automobile Spare Parts Market in Ilorin. Prior to multivariate analysis such as PCA, the normality of the data must be ascertained. Also, the Kaiser–Meyer–Olkin (KMO) and Bartlett's sphericity conditions must be satisfied in order to validate and interpret the PCA correctly^49,50^. KMO value between 0.65 and 1.0 is the acceptable range. In the study, the KMO and Bartlett's results were 0.795 and 206.5 (with the degrees of freedom df = 45 and p < 0.01) showing that PCA is useful in dimensionality reduction in this case. Table 8 shows the results of the PCA, which was based on the correlation coefficient matrix for the heavy metals in the study locations. The coefficient is assumed significant only when the value is greater than 0.30. Those with PCA coefficient lower than 0.30 were taken as having no significant contributions to the variations observed the soil samples^51^. From the table, two principal components were identified with Eigen-values greater than 1.0. PC1 with Eigen value of 5.984 accounts for 59.8% of the variation mainly from Mg, Mn, Zn, Cd, Pb, Cu, Fe, and Cr. PC2 with Eigen value of 1.277 accounted for 12.8% of the variation mainly from Mn, Ag and As. Based on the PCA results, two potential pollution sources were identified. PC1 clearly represents anthropogenic source based on the values of pollution indices presented in Table 8, while PC2 represent natural source such as the weathering of the parent rock.

**Table 8:** Principal component analysis (PCA) of heavy metal in soil of automobile spare parts market.

Heavy metal	PC1	PC2
Mg	0.837	− 0.061
Mn	0.574	0.608
Ag	− 0.136	0.805
Zn	0.887	0.163
Cd	0.852	0.279
Pb	0.692	0.142
Cu	0.951	0.116
Fe	0.965	0.008
As	0.190	0.579
Cr	0.869	0.276
Eigen value	5.963	1.277
% of variance	56.6	12.8
Cumulative %	56.6	72.4

### Result of pollution evaluation

The result of the modified enrichment factor (MEF), modified pollution index (MPI) and the anthropogenic metal is given in Table 9. The HMs exhibit different levels of enrichment ranging from minimal enrichment to significant enrichment in the order Zn > Mn > Mg > Cd > Cu > Cr > Ag > Fe > Pb > As. As, despite having concentration much higher than the global average, exhibited minimal enrichment indicating that its major source was geogenic with slight contribution from anthropogenic sources. This corroborates the results of the PCA analysis. Pb, Ag and Fe also exhibited minimal enrichment. While Cd and Cr have moderate enrichment, Zn, Mn and Mg exhibits significant enrichment. This reveals that the major source of these HMs is anthropogenic.

**Table 9:** Pollution evaluation.

	Mg	Mn	Ag	Zn	Cd	Pb	Cu	Fe	As	Cr
MEF	12.66	18.32	1.75	19.05	2.65	1.53	2.06	1.68	1.27	2.01
MPI	13.93	19.07	2.16	22.71	2.91	1.76	2.31	1.76	1.39	2.14
AM	1166.51	1732.41	75.00	1805.17	164.47	53.14	105.46	68.30	27.19	100.74

The HMs demonstrate slight to extreme pollution level. The levels of the pollution index (MPI) varies in similar manner as the enrichment factor i.e. Zn > Mn > Mg > Cd > Cu > Cr > Ag > Fe > Pb > As. This further confirm the agreement between the pollution evaluation parameters. The MPI reveals that the automobile spare part and recycling market is significantly polluted by Cd, Cu, Ag, Cr, Fe, Pb and As, and extremely polluted by Zn, Mn, and Mg. The results of the quantification of anthropogenic metal indicates that greater fractions of Zn, Mn, Mg, Cd, Cr, Cu, Ag, Fe and Pb in these soils are of anthropogenic origin. This further validates our earlier suggestion that the automobile spare part and recycling market is highly polluted and the pollution is more of anthropogenic than pedogenic and lithogenic sources. However, in spite of the fact that concentrations of As is multiple times higher than the global average, all the pollution evaluation indices corroborates the PCA analysis which reveals that significant amounts of As arises from lithogenic origin.

### Carcinogenic and non-carcinogenic hazards for the selected top soil samples

From the analysis of soil samples, Fe has the maximum mean concentration of 378 ppm and Ag has the minimum in the order Fe > Zn > Cr > Mg > As > Pb > Cu > Cd > Ag. For the Average Daily Intake via ingestion pathway, it was estimated for adult population and found that the ADI_ing_ ranges from 5.18E−4 to 4.11E−6 mg/kg-year with Fe contributing the highest via ingestion pathway (see Table 10). For ADI via inhalation pathway it was estimated and found that the ADI_inh_ ranges from 7.62E−8 to 6.04E−10 mg/kg-year with Fe still contributing the highest via inhalation pathway. For the ADI via dermal contact, it ranges from 6.96E−6 to 5.47E−8 with As contributing the highest. All the Hazard Index estimated are less than one (< 1) which is the standard set by USEPA (2001), with Arsenic contributing the highest via ingestion (see Table 10). This therefore means that there’s no probable non-cancerous effect. The total HI is also less than one. The Incremental Lifetime Cancer Risk was estimated as Cd (5.22E−6), Pb (3.26E−7), As (1.13E−4) and Cr (7.4E−5) with As contributing highest to cancer risk (see Table 11). As stated earlier, cancer risks greater than 1.00E−4 are considered high as they pose higher cancer risk while values below 1.00E−6 are considered not to pose any cancer risk to humans. Therefore, the acceptable range is between 1.00E−4 and 1.00E−6. Based on the Delphii method according to^36,37^, given by Table 2, the risk levels ranges between level I and level V. However, it should be noted that, the carcinogenic and non-carcinogenic risks reported in this present work may be underestimated values because the estimates did not capture intakes from food consumptions, other metals like mercury, nickel, cobalt etc. and the exposure parameters used (given in Table 12) were adopted from USEPA and so may not ineludibly represent a typical case of a Nigerian.

**Table 10:** Estimated annual dose intake of heavy metals in the top soil samples collected within the study site^31,35^.

HMs		Concentration (ppm)	ADI_ing_	ADI_inh_	ADI_derm_
Mg	Min–Max	42.00–64.00	6.00E−5 to 9.14E−5	8.82E−8 to 1.35E−7	2.39E−7 to 3.64E−7
	Mean	53.70	7.36E−5	1.08E−8	2.94E−7
Mn	Min–Max	58.00–72.00	8.29E−5 to 1.03E−4	1.22E−8 to 1.51E−8	3.30E−7 to 4.10E−7
	Mean	66.70	9.14E−5	1.34E−8	3.65E−7
Ag	Min–Max	2.00–5.00	2.86E−6 to 7.14E−6	4.20E−10 to 1.05E−9	1.14E−8 to 2.85E−8
	Mean	3.00	4.11E−6	6.04E−10	1.64E−8
Zn	Min–Max	105.00–210.00	1.50E−4 to 3.00E−4	2.21E−8 to 4.41E−8	5.98E−7 to 1.20E−6
	Mean	154.00	2.11E−4	3.10E−8	8.42E−7
Cd	Min–Max	8.20–12.00	1.17E−5 to 1.71E−5	1.72E−9 to 2.52E−9	4.67E−8 to 6.84E−8
	Mean	10.00	1.37E−5	2.02E−9	5.47E−8
Pb	Min–Max	25.00–37.00	3.57E−5 to 5.28E−5	5.25E−9 to 7.77E−9	1.43E−7 to 2.11E−7
	Mean	28.00	3.84E−5	5.64E−9	1.53E−7
Cu	Min–Max	18.00–28.00	2.57E−5 to 4.00E−5	3.78E−9 to 5.88E−9	1.03E−7 to 1.60E−7
	Mean	22.60	3.10E−5	4.55E−9	1.24E−7
Fe	Min–Max	340.00–410.00	4.86E−4 to 5.86E−4	7.14E−8 to 8.61E−8	1.94E−6 to 2.34E−6
	Mean	378.00	5.18E−4	7.62E−8	2.07E−6
As	Min–Max	35.00–50.00	5.00E−5 to 7.14E−5	7.35E−9 to 1.05E−8	1.09E−7 to 8.55E−6
	Mean	42.48	5.82E−5	8.56E−9	2.32E−7
Cr	Min–Max	90.00–122.00	1.29E−4 to 1.74E−4	1.89E−8 to 2.56E−8	5.13E−7 to 6.95E−7
	Mean	108.00	1.48E−4	2.18E−8	5.91E−7

**Table 11:** Estimated mean HI and ILCR of the heavy metals in soil samples collected within the study site^31,35^.

Heavy metals	THQ_ing_	THQ_inh_	THQ_derm_	HI	ILCR	Risk grades
Mg	–	–	–	–	–	–
Mn	1.99E−3	9.37E−4	1.98E−4	3.13E−3	–	–
Ag	–	–	–	–	–	–
Zn	7.03E−4	1.03E−7	1.40E−3	7.17E−4	–	–
Cd	1.37E−2		5.47E−3	1.90E−2	5.22E−6	Group I
Pb	1.10E−2	1.74E−6	2.91E−4	1.13E−2	3.26E−7	Group I
Cu	7.75E−2	1.13E−7	1.03E−5	7.85E−4	–	
Fe	6.00E−2	8.80E−6	2.39E−4	6.03E−2	–	
As	1.94E−1	2.84E−5	1.89E−3	1.94E−1	1.13E−4	Group V
Cr	4.90E−2	7.62E−4	9.85E−3	5.96E−2	7.40E−5	Group III

**Table 12:** Exposure parameters used in calculating the human health risks^31,38^.

S/N	Exposure parameters	Values	S.I unit
1	Ingestion rate IngR	100 for soil, 2 for water	mg/day for soil and L/day for water
2	Inhalation rate (InhR)	20	m^3^/day
3	Exposure frequency (EF)	365	day/year
4	Exposure duration (ED)	55	years
5	Body mass (BW)	70	kg
6	Time period of exposure (AT)	ED × 365	days
7	Particle emission factor (PEF)	1.36 × 10^9^	m^3^/kg
8	Exposed skin surface area (SA)	5700 for soil;	cm^2^
9	Adherence factor (AF)	0.07	mg/cm^2^-day
10	Dermal absorption factor (ABS)	0.001	
11	Chronic reference dose (RfD)	Ingestion RfD: Mn ($4.6 \times 10^{ - 2}$), Zn ($3.00 \times 10^{ - 1}$), Cu (4.00 $\times 10^{ - 2}$), Cr (3.00 $\times 10^{ - 3}$), Cd ($1.00 \times 10^{ - 3}$), Ni ($2.00 \times 10^{ - 2}$), Pb ($3.50 \times 10^{ - 3}$), As ($3.00 \times 10^{ - 3}$), Cd (1 × 10^–3^) Inhalation RfD:Mn ($1.43 \times 10^{ - 5}$), Zn ($3.00 \times 10^{ - 1}$), Cu (4.02 $\times 10^{ - 2}$), Cr (2.86 $\times 10^{ - 5}$), Ni ($2.06 \times 10^{ - 2}$), Co ($5.71 \times 10^{ - 6}$), Pb ($3.25 \times 10^{ - 3}$), As ($3.01 \times 10^{ - 4}$), Cd (5 $.70 \times 10^{ - 5}$) Dermal RfD: Mn ($1.84 \times 10^{ - 3}$), Zn ($6.00 \times 10^{ - 2}$), Cu (1.20 $\times 10^{ - 2}$), Cr (6.00 $\times 10^{ - 5}$), Cd (5 $.00 \times 10^{ - 4}$), Ni ($5.40 \times 10^{ - 3}$), Co ($1.60 \times 10^{ - 2}$), Pb ($5.25 \times 10^{ - 4}$), As (1.23 $\times 10^{ - 4}$)	mg/kg/day
12	Carcinogenic slope factor (SF)	Ingestion SF: As (1.5), Pb (8.5 $\times 10^{ - 3}$), Cr (0.5), Cd (0.38) Inhalation SF: Cr ($4.20 \times 10^{ - 1}$), Cd (6.30), Ni ($8.40 \times 10^{ - 1}$), As (1.51 $\times 10^{ - 1}$) Dermal SF: As (3.66)	(mg/kg/day)^−1^
13	Permeability constant (KP)	Pb, As, Cu (0.0001), Cr (0.002), Zn (0.006)	cm/h
14	Exposure time (ET)	0.58	hour/event

For the water samples within the study site, the Average Daily Intake via ingestion pathway estimated for adult population was presented in Table 13. It was found that the ADI_ing_ ranges between 2.00E−3 and 6.14E−2 mg/L-year with Zn contributing the highest. For the ADI via dermal contact, it was estimated and found that it ranges from 2.09E−8 to 6.41E−7 with Zn still contributing the highest. The Hazard Indices (HI) estimated for Cr, As and Pb are greater than 1 which is the recommended standard set by USEPA (2001), while others are within the recommended safe limit (< 1) (see Table 14). The total HI is 5.0171 which is far greater than one. This therefore means that the general populace should worry about the probable non-cancerous effect of these heavy metals. The Incremental Lifetime Cancer Risk (ILCR) was estimated and the values are: As (7.12E−2), Cr (2.93E−2), Cd (2.39E−3) and Pb (4.06E−4), with As contributing highest to the cancer risk followed by Cr, Cd and then Pb (see Table 14). Recall that cancer risks greater than 1.00E−4 are considered high since they pose higher cancer risk and values below 1.00E−6 are considered not to pose any cancer risk to humans, it follows that the cancer risks are very high and above the acceptable range. And based on the Delphii method according to^36,37^, given by Table 2, the risk levels ranges between level VI and level VII.

**Table 13:** Estimated annual dose intake of heavy metals in water samples collected within the study site.

Heavy metal		Concentration	ADI_ing_	ADI_derm_
Mg	Min–Max	0.71–1.50	2.0E−2 to 4.29E−2	2.12E−7 to 4.47E−7
	Mean	1.09	3.11E−2	3.25E−7
Mn	Min–Max	0.06–0.08	1.71E−3 to 2.29E−3	1.79E−8 to 2.39E−8
	Mean	0.07	2.00E−3	2.09E−8
Ag	Min–Max			
	Mean	–	–	–
Fe	Min–Max	1.30–2.50	3.71E−2 to 7.14E−2	3.88E−7 to 7.46E−7
	Mean	1.75	5.00E−2	5.22E−7
Zn	Min–Max	1.50–3.00	4.29E−2 to 8.57E−2	4.47E−7 to 8.95E−7
	Mean	2.15	6.14E−2	6.41E−7
Cd	Min–Max	0.18–0.25	5.14E−3 to 7.14E−3	5.37E−8 to 7.46E−8
	Mean	0.22	6.29E−3	6.56E−8
As	Min–Max	0.58–2.22	1.66E−2 to 6.34E−2	1.73E−7 to 6.62E−7
	Mean	1.66	4.74E−2	4.95E−7
Cr	Min–Max	1.40–2.70	4.00E−2 to 7.71E−2	4.18E−7 to 8.05
	Mean	2.05	5.86E−2	6.12E−7
Pb	Min–Max	1.20–2.00	3.43E−2 to 5.71E−2	3.58E−7 to 5.97E−7
	Mean	1.67	4.77E−2	4.98E−7
Cu	Min–Max	1.10–2.18	3.14E−2 to 6.23E−2	3.28E−7 to 6.50E−7
	Mean	1.38	3.94E−2	4.12E−7

**Table 14:** Estimated mean HI and ILCR of the heavy metals in water samples collected within the study site.

S/N	Heavy metal	THQ_ing_	THQ_derm_	HI	ILCR	Risk grades
1	Mg	–	–	–	–	–
2	Mn	0.0435	1.13E−5	0.0435	–	–
3	Ag	NA	NA	NA	–	–
4	Fe	0.5556	5.80E−6	0.5556	–	–
5	Zn	0.2048	1.07E−5	0.2048	–	–
6	Cd	0.6286	6.56E−3	0.6351	2.39E−3	Group VI
7	As	1.5810	4.03E−3	1.5850	7.12E−2	Group VII
8	Cr	1.9524	1.02E−2	1.9626	2.93E−2	Group VII
9	Pb	1.3633	9.49-4	1.3642	4.06E−4	Group VI
10	Cu	0.0986	3.43E−5	0.0986	–	–

**Figure 4:**
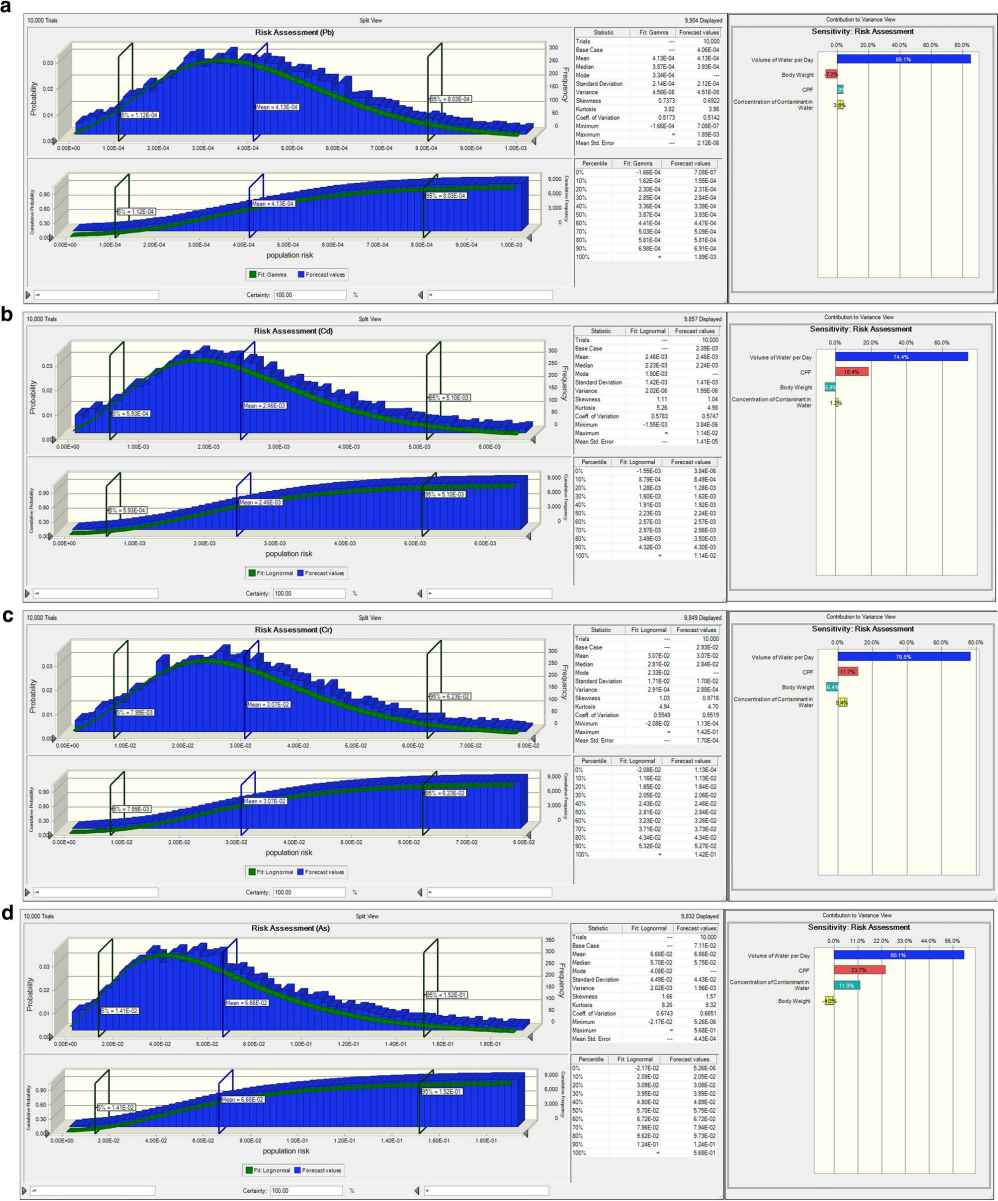
Cumulative probability plot of the Cancer risks for the water samples from the automobile spare part and recycling park: (**a**) Lead (Pb), (**b**) Cadmium (Cd), (**c**) Chromium (Cr) and (**d**) Arsenic (As).

Since ingestion pathway is the dominant exposure route, and our major concerns are the risks posed by the known human carcinogens, the Monte Carlo simulation was used to estimate the Incremental Lifetime Cancer Risk (carcinogenic risk assessment) for the ingested water samples. The Monte Carlo simulation model was run for 10,000 total trials. The mean, 5th and 95th percentiles of the ILCR distribution were determined. The result of the MCS is given in Table 15 and Fig. 4a–d. According to the mean, P 5% and P 95% cumulative probability due to ingestion of water, the ILCR which is employed for carcinogenic risk assessment in this work is above the safe region of 1.00E−6 and 1.00E−4 recommended by USEPA. According to the P 95% cumulative probability, As (1.52E−1) posed the highest risk followed by Cr (6.23E−2), Cd (5.10E−3) and then Pb (8.03E−4). The sensitivity chart from the MCS reveals that the volume of ingested water ranks highest (which agrees with the estimated ADI_ing_ for the water samples) followed by the slope factor (CF), Concentration of the metals in water and then the body weight, which is negative.

**Table 15:** Summary of the Monte Carlo simulation.

Heavy metal	5%	Mean	95%
Pb	1.12E−4	4.13E−4	8.03E−4
Cd	5.93E−4	2.46E−3	5.10E−3
Cr	7.99E−3	3.07E−2	6.23E−2
As	1.41E−2	6.66E−2	1.52E−1

## Conclusion

The concentrations of the heavy metals in the samples of soil and water collected within the automobile spare part and recycling market are much higher than the ones recorded outside the market. The concentration of the heavy metals at the market decreases with an increase in depth from 0 to 50 cm and appears to be stable below 50 cm of the soil depth. This follows that the enhanced level of the heavy metals at the study site is majorly due to the activities at the automobile spare part and recycling park (i.e. vehicular discharges and dusts, painting, wielding, poor disposal of spare part etc.). This revelation was corroborated by all the pollution evaluation indices (MEF, MPI and AM) and the PCA analysis. While all the Hazard Index (HI) estimated for the soil samples are less than one (< 1) which is the standard set by USEPA, the Hazard Indices (HI) estimated for Cr, As and Pb are greater than 1 for the water samples within the automobile spare part and recycling park. The Incremental Lifetime Cancer Risk levels ranges between level I and level V for the soil samples and ranges between level VI and level VII for the water samples within the study site. This follows that the cancer risks are very high and above the acceptable range for the water exposure route. Similarly, according to the mean, P 5% and P 95% cumulative probability due to ingestion of water using the Monte Carlo simulation, the carcinogenic risk assessment in this work is above the acceptable range of 1.00E−6 and 1.00E−4 recommended by USEPA.

It therefore follows that the study area is polluted because of the anthropogenic activities at the automobile spare part and recycling park. Finally, it is recommended that a more robust work that considered mass flux/mass discharge concept models to estimate the dilution of the heavy metal transport mechanism be carried out in the future.
